# Debate: Young people are living in unprecedented times—too much chaos or too little resilience? Beyond the ‘chaos’ storyline—Modernising resilience frameworks and mental health care for children and young people in a neurodiversity inclusive digital world

**DOI:** 10.1111/camh.70090

**Published:** 2026-03-18

**Authors:** Julia Dray

**Affiliations:** ^1^ Graduate School of Health, Faculty of Health University of Technology Sydney Sydney Australia

**Keywords:** resilience, neurodiversity, artificial intelligence, children and young people, mental health

## Abstract

Children today, in 2026, are growing up in a world that feels busy, fast and sometimes overwhelming. This raises questions: is the world too chaotic, are children and young people not resilient enough or could it be that our support systems have not kept up with the ‘chaos’? Our current world is characterised by rapid technological change, algorithmically amplified social pressures, evolving global uncertainties translating into new reasons for worry and anxiety, and increased prevalence of mental health problems in children and young people globally. They are online at school, at home and all but constantly in the world around them. A modern resilience agenda must therefore prioritise safety‐by‐design online, neurodiversity‐affirming educational and digital environments and expanded care capacity, supported by learning health systems that iterate quickly as conditions change. Our big picture systems and infrastructures have not kept pace and evolved at speed alongside digital advances and AI‐driven environments. Consideration must be given to how digital and AI‐enabled mental health tools can mitigate service gaps when demand exceeds workforce capacity, while emphasising and ensuring ethical, inclusive, accessible and developmentally attuned design. Modernising systemic supports is essential for cultivating youth resilience and mental health in an increasingly complex digital world.

The current and future generations of children and young people are and likely will continue to grow up in a world often described as turbulent, unpredictable and overwhelming. Prominent in our day to day are rapid technological shifts, global crises, climate change, political division, economic instability and the accelerating integration of artificial intelligence. Examples include voice‐controlled home assistants, AI companions and conversational agents, AI tutors in every pocket, and AI‐curated social media threads. These sweeping changes have not occurred in isolation. Globally, they are mirrored by a marked rise in prevalence of mental health difficulties, self‐harm, suicide and the use of child and youth mental health services and hospital admissions in recent years (WHO, 2021). These services remain essential for supporting the well‐being and development of children and young people yet demand now far exceeds capacity. This imbalance is contributing to widespread staff burnout and workforce shortages (Cummings et al., 2023), longer wait times, more severe and complex presentations and growing numbers of young people whose mental health needs remain unmet (Cummings et al., 2023).

Against this backdrop, the debate question—whether young people face *too much chaos* or whether *too little resilience* has been built around them—invites deeper examination. Importantly, and from a lived experience perspective of a mum of two tiny humans of today's generation, the chaos itself is not new; the speed, intensity and mediated nature of it, however, are largely unprecedented. Today's children and young people confront not only traditional developmental changes and challenges but also an information environment, where algorithms influence social belonging, identity, emotion and behaviour in real time, and AI‐facilitated platforms exist in almost every facet of their day (and sometimes night). Framing the problem as insufficient individual resilience risks obscuring a more pressing truth: resilience at a family, education, mental health service, digital platform design and policy level has failed to evolve at the same pace as the *chaos* young people now navigate. Young people are not inherently less resilient; rather, at a systemic level, the scaffolding required to support their adaptive capacity has not been re‐engineered for a digital and AI‐saturated era. When these ecologies lag behind the AI‐shaped context children and young people inhabit, distress rises.

To understand this tension, it is necessary to acknowledge the ways in which the digital world amplifies developmental vulnerabilities, and the ways in which innovative and accessible mental health solutions can harness new technologies to better support the mental health of children and young people (Dray & Symons, 2025; Ramshaw et al., 2023). Periods of childhood including adolescence are characterised by increased sensitivity to rewards, a strong focus on social connections, active identity development and significant emotional variability, all of which are grounded in robust neurodevelopmental research (Venticinque, McMillan, & Guyer, 2024). By providing constant social feedback and algorithmically curated comparison, AI‐driven loops may intensify social comparison, emotional reactivity, identity formation and exposure to a wider and often harsher peer audience. These mechanisms may contribute to a sense of ‘chaos’ complicating developmental adaptation and reshaping pathways to mental health outcomes, both positively and negatively.

Innovative mental health solutions are increasingly leveraging emerging technologies to address gaps in care and improve outcomes for children and young people (Dray & Symons, 2025). For example, AI‐powered chatbots and digital companions can provide immediate, low‐intensity support, helping users manage anxiety, access resources and practise coping skills outside of traditional clinical settings (Dray & Symons, 2025). These tools are often available 24/7, breaking down barriers related to location or clinical hours, cost and stigma, and if developed responsibly and ethically, have the potential to triage users to higher levels of care when needed (Dray & Symons, 2025). These solutions can also allow children and young people to engage with therapeutic content in ways that suit their unique cognitive and emotional profiles, including through neurodiversity‐affirming features, and fostering engagement and sustained use. Machine learning and data analytics can also play a vital role by identifying patterns of mental health risk and resilience from user interactions (or sometimes absence of), and thus have the potential to enable early intervention and personalised support (Dray & Symons, 2025). If implemented ethically and inclusively, these technologies could extend the reach of mental health services, reduce wait times and ensure that interventions are responsive to the diverse needs of today's digitally connected children and young people.

What about resilience? Traditional resilience models emphasise protective factors such as emotional regulation, coping skills and secure relationships (Dray, 2021), and these continue to matter. However, the resilience needed in an AI‐shaped digital era includes additional dimensions: digital literacy, information sensibility, critical algorithmic awareness, the ability to contextualise online experiences, and the capacity to manage constant cognitive load (Hassoun et al., 2025; Qi & Yang, 2024). Our children and young people now also need digital resilience: the ability to recognise, manage and recover from online risks (Qi & Yang, 2024). Furthermore, studies highlight how adolescents interpret information collaboratively, often relying on peers, shared identities and online communities to make sense of the world (Hassoun et al., 2025). This suggests that resilience is no longer solely an individual attribute but increasingly a *networked* one, shaped through digital social processes. Hence, challenges arise when the systems around young people continue to rely on outdated models.

Neurodiversity adds further complexity to this picture. Resilience in neurodivergent populations is highly variable, multi‐dimensional and inconsistently conceptualised across the literature (Black et al., 2024); the latter is still an unresolved difficulty identified in the broader field of child and adolescent resilience (Dray, 2021). Further, despite decades of research, the field has yet to establish clear mechanisms of how specific protective factors translate into better mental health (Dray, 2021), or how resilience pathways differ for neurodivergent youth whose sensory, cognitive and social profiles may interact with digital contexts in distinct ways (Black et al., 2024; Hassoun et al., 2025). Mainstream resilience frameworks rarely account for the sensory, cognitive and communicative differences that shape how neurodivergent children and young people navigate the world, resulting in systemic mismatches (Black et al., 2024). Given the central role of online spaces in identity, belonging and participation, the debate must consider how digital systems reinforce or undermine neurodivergent resilience pathways, as well as how the social and structural supports surrounding them do.

This leads back to the broader issue: youth mental health systems themselves are under unprecedented strain, and the rising prevalence of mental health concerns among adolescents is well documented (WHO, 2021). As noted, digital innovations offer a partial answer to the systems‐level resilience and mental health care gap: digital tools can complement, augment, and extend human‐led child and youth mental health care when demand overwhelms supply. The risk is that digital solutions become quick fixes rather than meaningful supports, especially if deployed without responsible and ethical oversight, cultural responsiveness, accessibility and feasibility considerations, or co‐design and integration with human professionals and evidence‐based infrastructures (Dray & Symons, 2025). Digital mental health tools can only foster resilience when they are designed and deployed as part of broader, coherent ecosystems where AI enhances, rather than replaces, human connection and expertise. This requires investment in technology development, governance frameworks, training for clinicians and educators, and participatory design that centres the lived experiences of diverse children, young people, their families and health professionals (Dray, 2021; Dray, Palmer, & Banfield, 2024). Neurodivergent children, young people, and professionals should be included in the design and evaluation of digital resilience and mental health strategies, ensuring interventions recognise diverse processing styles rather than impose neurotypical norms.

Ultimately, the question at the heart of the debate is not whether chaos has increased—it has—nor whether young people have too little resilience—they do not. The real problem is that we are asking young people to navigate unprecedented complexity with resilience and mental health infrastructures that were never built for this moment in time. Systemic, relational, and technological resilience must be strengthened in tandem if current and future generations of children and young people are to thrive. The next generation of resilience science must therefore prioritise rigorous, mechanism‐based trials, improved measurement of protective factors, and analytic attention to differential effects, while integrating an understanding of how algorithmic curation, AI companions, and ubiquitous online social feedback amplify developmental sensitivities and impact mental health. Only by uniting resilience and mental health research with contemporary digital realities, and ensuring that interventions are co‐designed with the people they are intended to help and inclusive of neurodiversity, can we build multi‐layered resilience infrastructures that genuinely support the mental health of children and young people in the world they inhabit today.

## Funding information

The study was funded by a 2024 University of Technology Sydney (UTS) Disability Access and Inclusion Grant, awarded to the lead author as the Chief Investigator (CI).

## Conflict of interest statement

The author declares no competing interests.

## Ethics statement

No ethics approval was required for this debate article.

## Data Availability

Data sharing is not applicable to this article as no data sets were generated or analysed for this debate article.
